# Little Impact of NaCl Reduction in Swiss-Type Cheese

**DOI:** 10.3389/fnut.2022.888179

**Published:** 2022-06-16

**Authors:** Valérie Gagnaire, Xavier Lecomte, Romain Richoux, Magali Genay, Julien Jardin, Valérie Briard-Bion, Jean-René Kerjean, Anne Thierry

**Affiliations:** ^1^UMR STLO, INRAE, Institut Agro, Rennes, France; ^2^CALBINOTOX, Université de Lorraine, Nancy, France; ^3^Actalia Dairy Products, Rennes, France

**Keywords:** Emmental cheese, salt, proteolysis, lactic acid bacteria, *Lactobacillus helveticus*, cell-envelope proteinase, casein, hard cheese

## Abstract

Reducing salt intake can mitigate the prevalence of metabolic disorders. In fermented foods such as cheeses, however, salt can impact the activity of desirable and undesirable microorganisms and thus affect their properties. This study aimed to investigate the effect of salt level on Swiss-type cheese ripening. Since proteolysis is a major event in cheese ripening, three strains of *Lactobacillus helveticus* were selected on the cell-envelope proteinase (CEP) they harbor. Their proteolytic activity on caseins was studied at six salt levels (0–4.5%) at pH 7.5 and 5.2. Swiss-type cheeses were manufactured at regular, increased, and decreased salt concentrations, and characterized for their composition and techno-functional properties. *L. helveticus* strains possessed and expressed the expected CEPs, as shown by PCR and shaving experiments. The two strains of *L. helveticus* that possessed at least the CEP PrtH3 showed the greatest proteolytic activity. Casein hydrolysis *in vitro* was similar or higher at pH 5.2, i.e., cheese pH, compared to pH 7.5, and slightly decreased at the highest salt concentrations (3.0 and 4.4%). Similarly, in ripened cheeses, these *L. helveticus* strains showed 1.5–2.4 more proteolysis, compared to the cheeses manufactured without *L. helveticus*. Regarding the salt effect, the 30% salt-reduced cheeses showed the same proteolysis as regular cheeses, while the upper-salted cheeses showed a slight decrease (−14%) of the non-protein fraction. The microbial and biochemical composition remained unchanged in the 30%-reduced cheeses. In contrast, *Propionibacterium freudenreichii*, used as ripening bacteria in Swiss cheese, grew more slowly in upper-salted (1.14%, w/w) cheeses, which induced concomitant changes in the metabolites they consumed (−40% lactic acid) or produced (fivefold decrease in propionic acid). Some cheese techno-functional properties were slightly decreased by salt reduction, as extrusion (−17%) and oiling off (−4%) compared to regular cheeses. Overall, this study showed that a 30% salt reduction has little impact in the properties of Swiss-type cheeses, and that starters and ripening cultures strains could be chosen to compensate changes induced by salt modifications in Swiss-type and other hard cheeses.

## Introduction

The Western diet, rich in saturated fats, refined carbohydrates, and salt, has been linked to the increased prevalence of metabolic disorders. Reducing salt intake is among the most cost-effective interventions to reduce the burden of non-communicable diseases such as cardiovascular diseases (1). The World Health Organization proposed in 2012 to reduce sodium intake by 30% by 2020, so as to reach 2 g/day (i.e., 5 g of salt/day) salt intake (2). However, salt in food influences not only its nutritional qualities but also its safety, sensory, and other properties. Salt, together with pH, water activity, and redox potential, shows efficacy against pathogenic and spoilage microorganisms (3, 4). Salt reduction topic has been addressed in the frame of the TERIFIQ (Combining Technologies to achieve significant binary Reductions in Sodium, Fat and Sugar content in everyday foods whilst optimizing their nutritional Quality) European project (5). Amongst food products, fermented foods such as cheeses are a special case because the desirable microbial and enzymatic activities in these products can also be affected by changes in salt content, thus leading to dramatic changes within the product. For example, in cheese, a decrease in salt content up to 50% can lead to an increase in food pathogen or spoilage bacteria as *Clostridium botulinum* (6), *Clostridium tyrobutyricum* (7), and *Listeria monocytogenes* (8). Salt decrease can also induce changes in the overall physico-chemical, microbiological, and sensory qualities of soft and semi-hard cheeses (9), as well as the proteolytic and cooking properties of Cheddar cheese (10). It is therefore important to investigate the effect of salt on all the factors that are susceptible to influence the global quality of the product, including, in the case of fermented foods, the spontaneous microbiota or the starter cultures, and their main enzymes.

Lactic acid bacteria (LAB) participate in the manufacture of almost all fermented foods (11, 12). *Streptococcus thermophilus* is used along other LAB starters for its fast-acidification activity in milk. *Lactobacillus helveticus* is associated with *S. thermophilus* to complete milk acidification. It is also used for its high proteolytic activity, which can result in diverse benefits: it accelerates flavor development (13), reduces bitterness (14), contributes to cheese techno-functional properties (15–17), and can also generate bioactive peptides (18). The proteolytic system of LAB is complex, and includes cell-envelope proteinases (CEPs), transport system, and intracellular peptidases. Six main types of CEPs have been identified in LAB: PrtP in *Lactococcus lactis*, PrtS in *S thermophilus*, and PrtH, PrtR, PrtB, and PrtL found in *L. helveticus*, *Lactobacillus rhamnosus, Lactobacillus delbrueckii* subsp. *bulgaricus*, and subs*p. lactis*, respectively (19). Most of the LAB possess only one CEP but *L. helveticus* displays up to four CEPs depending on the strain (20). *L. helveticus* strains possessing different numbers of CEP genes were previously characterized for their ability to differently hydrolyze *in vitro* β- and α_s1_-caseins (21). However, the knowledge is scarce on the expression and the activity of these CEPs under cheese salt and pH conditions. The level of salt in cheese is typically in the range 1.5 and 3% (w/w), as for Cheddar cheese, but varies over a large range, from ∼0.5% (w/w) for Swiss-type cheese to ∼4.5% or even higher for Blue cheese and some other cheeses (4), while cheese pH ranges from 4.5 to 7.0 (4). The optimal pH of CEP activity ranges from 5.5 to 7.0 (19).

The aim of this study was to investigate how the salt level affects the ripening and composition of Swiss-type cheese, with a focus on the proteolytic activity of LAB strains. For this, several LAB strains of *L. helveticus*, selected for harboring different CEPs, were first characterized for the presence of proteinases on the cell surface, by a shaving approach. Their proteolytic activity on α_s1_- and β-caseins was studied *in vitro* at different NaCl levels, and then in Swiss-type-type cheese at regular, increased and decreased salt concentrations. Cheeses were globally characterized for their microbial and physico-chemical composition and their techno-functional properties.

## Materials and Methods

### Bacterial Strains and Growth Conditions

Three *L. helveticus* strains were selected for their content in CEP genes according to previous studies (21–23) (Table 1). They were cultivated at 42^°^C for 24 h without shaking in de Man Rogosa and Sharpe broth (MRS, Difco, Fisher scientific, France) (24) or in 10% (wt/vol) reconstituted low heat skim milk powder (21). Three successive precultures were performed with an inoculation level of 1%. The strains were inoculated in the cheese to obtain 10^5^ colony-forming units (CFU)/mL of cheese milk per strain.

**TABLE 1:** *Lactobacillus helveticus* and *Streptococcus thermophilus* strains used in this study and CEP genes identified in their genome sequences.

Strains (other name)^a^	Cell-envelope proteinase genes^b^					Origin	Reference	Use in this study
	*prtH*	*prtH2*	*prtH3*	*prtH4*	*prtS*			
***L. helveticus***								
CIRM-BIA103 (CNRZ32)	+	+	+	+	−	Cheese	(21)	*In vitro/*in cheese
CIRM-BIA99 (ITGLH77)	−	−	+	−	−	Cheese	(56)	*In vitro/*in cheese
RO052	−	−	−	+	−	Cheese	(56)	*In vitro*
***S. thermophilus***								
PALITG ST88	−	−	−	−	+	Cheese		In cheese

*^a^Collections, CIRM-BIA: a Biological Resource Center dedicated to bacteria for food products, INRAE Rennes, France, https://collection-cirmbia.fr/, PALITG: Laboratoires Standa, Caen, France; L. helveticus R0052 was kindly provided by Dr. Thomas Tompkins from the Rosell Institute, Montreal, QC, Canada, PALITG ST88 was from Laboratoires Standa, Caen, France.*

*^b^+ Presence; −Absence.*

Enumerations were performed on MRS or LM17 agar (1.5%) plates, at 42^°^C × 48 h under anaerobic and micro-aerophilic conditions for *L. helveticus* and *S. thermophilus*, respectively (25). For cheese samples, 10 g of grated cheeses, at 1, 8, 20, 34, and 48 day of ripening were dispersed in 90 mL of sodium citrate (20 g.L-1) in bag and crushed in the Smasher (AES laboratoire, Bruz, France) as previously described (26).

### *Lactobacillus helveticus* Strain Characterization

#### Genetic Characterization

The presence of CEP genes was studied for the three *L. helveticus* strains by PCR amplifications according to the conditions used by Lecomte et al. (27) and by using the specific CEP primers described by Broadbent et al. (23). Four fragments of different lengths were amplified corresponding to genes *prtH* (fragment of 1.332 kb), *prtH2* (4.042 kb), *prtH3* (0.357 kb), and *prtH4* (3.386 kb).

#### Growth and Acidification Rate in Milk

The growth of LAB strains in 10% (w/v) reconstituted skimmed milk was followed by monitoring the acidification with CINAC 3 system (Ysebaert, France). A mean acidification rate between pH 6.0 and 5.0 (pH unit/h) was calculated from this portion of the pH curve, considered as linear, as previously done (28). LAB were enumerated just after inoculation and at the stationary phase.

#### Surface Protein Identification by Shaving and Tandem Mass Spectrometry

A shaving of bacterial cells, i.e., a tryptic digestion of surface proteins, was performed according to Lecomte et al. (27). Briefly, three *L. helveticus* strains were grown at 42^°^C in 10% reconstituted skim milk to an OD_480 nm_ ∼2, corresponding the exponential growth phase. The absorbance at 480 nm was measured after clarification of the milk with 0.2% EDTA (pH 12). Cells were washed and concentrated to OD_650 nm_ = 30 in PBS (Phosphate-buffered saline) containing 5 mM DL-dithiothreitol (Sigma-Aldrich, St Quentin Fallavier, France), adjusted to pH 8.5. Five μg of sequenced grade trypsin (Promega, Charbonnières, France) were added to 500 μL of the concentrated cell suspension. Negative controls contained the same mixture except trypsin. Cells were incubated for 1 h at 37^°^C under shaking (180 rpm). Supernatants were harvested by centrifugation at 10,000 g, 10 min at room temperature and filtrated through a 0.45 μm filter (Millex PVDF, 13 mm, Millipore, Molsheim, France). Each supernatant was divided in two aliquots: in the first, the reaction was stopped by adding 15 μL of 10% (v/v) trifluoroacetic acid (TFA) (Sigma Aldrich) and samples were frozen at −20^°^C. In the second, supernatants were further incubated overnight at 37^°^C under gentle shaking (100 rpm) in the presence of 1 μg of additional trypsin then the reaction was stopped as previously by TFA addition. All samples were stored at −20^°^C prior to mass spectrometry analyses.

Mass spectrometry (MS) experiments were performed as previously described (27). To identify peptides, MS and MS/MS data were analyzed using the X! Tandem pipeline developed by the PAPPSO platform (29). The search was performed against a database composed of the taxonomy Bacilli from www.uniprot.org (Taxon identifier: 91061) to which was added the deduced sequences of the four CEP of *L. helveticus* CIRM-BIA103 (PrtH, PrtH2, PrtH3, and PrtH4), and PrtH3 from *L. helveticus* CIRM-BIA99. Database search parameters were specified as follows: trypsin cleavage was used and the peptide mass tolerance was set to 10 ppm for MS and 0.05 Da for MS/MS. Oxidation of methionine was selected as a variable modification. Peptides including several Lys or Arg in the sequence were allowed during the “refinement” process of X!tandem. For each peptide identified, a minimum score corresponding to an e-value below 0.05 was considered as a prerequisite for peptide validation. The identified proteins were conserved when at least two specific peptides were identified.

### Assessment of α_s1_- and β-Caseins Hydrolysis

After growth in milk (OD_480 nm_ = 2, before milk coagulation), cells were washed first in tri-sodium citrate solution 0.25 M (Carlo Erba, Val de Reuil, France) and centrifuged at 8,000 g for 10 min at 4^°^C and washed twice in Tris Buffer Salt at 150 mM NaCl, then twice in 50 mM Tris–HCl pH 7.5. Cells were suspended at OD_650 nm_ = 20 in 50 mM Tris buffer pH 7.5 + 20 mM CaCl_2_, or potassium lactate 50 mM pH 5.2 + 20 mM CaCl_2_. Cells were diluted to OD_650 nm_ = 10 with the same buffers containing different NaCl concentrations to have final NaCl concentrations of 0, 0.5, 0.75, 1.5, 3, and 4.5% NaCl (w/v). The α_s1_- and β-caseins, prepared as described in Sadat-Mekmene et al. (21), were added to each cell suspension at 5 mg.mL^–1^ final concentrations. Samples were collected after 0.25, 0.5, 1, 2, 3, and 15 h of hydrolysis. The reaction was stopped with 3 μl of 100 mM phenylmethylsulfonyl fluoride (PMSF). Cell pellets were eliminated after centrifugation (6,000 *g* for 10 min at 4^°^C), and the supernatant was filtered through a 0.45 μm-pore-size PVDF filter and was stored at −20^°^C. Different controls were performed: (i) incubation of the cells in absence of α_s1_- and β-caseins to check cells lysis at NaCl levels of 0, 1.5, and 4.5% in the Tris or lactate buffers at 3 times of incubation and (ii) casein control in the absence of cells at NaCl levels of 0, 1.5, and 4.5%.

### Tris Tricine SDS-PAGE

Hydrolysis of purified α_s1_- or β-caseins by strains of *L. helveticus* was monitored by Tris-tricine SDS-PAGE at different times (from 0.25 to 15 h) as previously described (21). The density of α_s1_- and β-casein bands was measured and plotted against time, and the slope of these curves between T0 (casein control without hydrolysis) and the time of disappearance of casein bands was used to calculate a rate of casein hydrolysis (% casein hydrolyzed/min) for each strain, pH, and NaCl concentration.

### Cheese Manufacture

Nine types of cheese were manufactured according to a 3^2^ experimental design. The two factors were the NaCl concentration and the *L. helveticus* strain used as starter, each at three levels. Concerning salt, we tested a regular, a low and a high salt concentration. The regular concentration N1 was 0.45% (w/w), i.e., 1.2% salt/moisture. The low level, N0.7, corresponded to a 30% reduction, in accordance to the WHO recommendations (2), while the high level, N2.5, was a 2.5-fold higher salt content to mimic the concentration near the cheese rind or the one present in other hard cheese varieties (4). Concerning *L. helveticus* strains, we chose the two most proteolytic strains studied *in vitro*, CIRM-BIA103 and CIRM-BIA99, and added a control without *L. helveticus*. These *L. helveticus* strains were associated with the same commercial *S. thermophilus* strain (PALITG ST88 from Laboratoires Standa, Caen, France). Raw milk was purchased in a local herd (EARL Le Marchand, Pacé), pasteurized at 74^°^C for 20 s in an electric tubular device (Actijoule, Actini, Evian, France) and then skimmed at 55^°^C. The pasteurized skim milk was then microfiltered on an AlfaLaval pilot equipped with a Membralox ceramic gradient of porosity Membrane (Pall-Exekia, Tarbes, France) with 1.4 μm pore size (running conditions: 52^°^C, volumetric concentration factor 20, transmembrane pressure: 1.2 bar), to remove residual bacteria and somatic cells. The cream was heat-treated in Actijoule at 120^°^C for 20 s. The protein and fat content of the skim milk microfiltrate and the cream composition were determined by IR-analyzer (Lactoscope, Deft, The Netherlands) and the fat/casein ratio of the cheesemilk was adjusted to 1.15. Each batch of cheesemilk was stored at 4^°^C for 12, 36, or 70 h for cheese making. A single strain of thermophilic lactobacilli was used for each week of cheesemaking experiment in order to avoid cross-contamination and cheeses with an upper, regular and lower salt content were made with 12, 36, and 70 h stored milk, respectively.

Three cheeses were made in parallel in three stainless steel double jacked 200 L cheese vats equipped with an automate (Roussel Inox, Creissels, France). The milk (100 kg per vat) was supplemented with calcium (20 mL/vat of a 47% CaCl_2_ solution, Maxical, Chr Hansen, Arpajon, France), warmed at 32^°^C and inoculated with starters: (i) 0.12% of a culture of *S. thermophilus* PALITG ST88 grown at 42^°^C for around 4 h in Marstar 412 A medium (Danisco-Dupont, Dangé Saint-Romain, France), (ii) 0.1% of a culture of *L. helveticus* grown on Phagex Lb medium (Laboratoires Standa) at 42^°^C for 6–8 h to reach a lactic acid concentration of 6 g/L and (iii) 1 g/vat of freeze dried *Propionibacterium freudenreichii* PALITGP20 (Laboratoires Standa, Caen, France). After 30 min of milk ripening, the milk (pH 6.60 and 32^°^C) was coagulated using 20 mL of recombinant bovine chymosin (Chymax plus 200 IMCU/mL, Chr Hansen). When the targeted firmness of the gel was reached, the coagulum was cut with blades.

In order to keep the final dry matter constant and to get similar gross composition for all cheeses despite their different salt content after brining and the different LAB combinations used as starters, several preliminary experiments were performed (data not shown) and the manufacture recipe was modulated as described below.

First, to increase the moisture at day 1 for the upper salt level cheeses, both gel firmness and cutting diagram were adjusted depending on the targeted salt content, with a higher size of curd grain for the upper salt level cheeses. To avoid pH differences at day 1 cheese, deionized water at 33^°^C (10 L) was also added after cutting the cheeses with upper salt content to increase moisture before brining. The curd grains and whey of the cheeses with lower salt content were stirred before cooking for 10 min and all cheeses were heated to 53^°^C in 25 min and cooked at this temperature for 10 min for upper salt or 20 min for regular and lower salt level cheeses. The curd draining was performed by gravity into a stainless-steel macro perforated mold for pre-pressing at 10 g/cm^2^ for 30 min. Thereafter, the pre-pressed curd was cut into five parts of around two kg each, which were placed in micro-perforated plastic molds (18 cm diameter) for pressing at 100 g/cm^2^ for one h in a thermostated chamber (35^°^C, 90% RH) and then the acidification step was performed for 3 h at 45^°^C and the next for 16 h at 35^°^C. The cheeses were demolded and cooled in the brining room (13^°^C) for 4 h. One cheese block was withdrawn for analysis at day 1 while the four remaining cheese blocks were salted in saturated brine under stirring at 12^°^C for either 75 min, 4.5 h or 20 h according to the final salt content targeted. Cheeses were soaked by 1,000 ppm natamycin solution (Mycopim, Laboratoires Standa) before being packed under vacuum in semi-permeable and thermo-retractable ripening bags (BK1L, Cryovac, Epernon, France). They were ripened 20 days at 12^°^C and then 18 days at 23^°^C. Two kg of cheese were withdrawn for analyses after 8, 20, 34, and 48 days of ripening, and sampled at the middle of the radius with a cheese borer.

Cheeses with *S. thermophilus* as a sole lactic starter were manufactured according to the process described above except the addition of 50 g of edible lactose powder (Lactalis Ingredients, Bourgbarré, France) per kg skim milk before microfiltration, in order to reach a similar pH in cheese at day 1.

### Cheese Analysis

Dry matter (DM) was determined by drying method according to IDF standard n°4A. Fat content was determined using the acido-butyrometric method (30). Fat in Dry Matter (FDM) and moisture in non-fat substance (MNFS) were calculated as follows: FDM = 100 × (fat/DM), MNFS = 100 × (100-DM)/(100-fat). Calcium was measured by the complexometric method of Pearce (31). NaCl was calculated from chloride determination by potentiometric method with a Chloride Model 926 (Sherwood Scientific Ltd., Cambridge, United Kingdom). Total nitrogen (TN) was determined using the Kjeldahl method and total nitrogen matter calculated as TN × 6.38 (32).

Cheese proteolysis was assessed by determination of pH 4.6-soluble nitrogen (i.e., non-casein nitrogen, NCN) and 12% TCA-soluble nitrogen (i.e., non-protein nitrogen, NPN) according to Gripon et al. (33). Cheese water-soluble extracts were prepared by adding 40 g of distilled water to 10 g of grated cheese (ratio 1:5) (34). The mix was blended using an ultra turrax (IKAT18^®^ basic, Staufen, Germany) for 5 min at 20,500 rpm and further stirred for 30 min at 40^°^C. Water-soluble extracts were recovered after centrifugation (6,000 g for 30 min at 4^°^C) and fat elimination. They were filtered on Whatman filter paper 40 at 4^°^C and frozen at −20^°^C until further use.

Casein hydrolysis was evaluated by urea-PAGE from the pH 4.6-insoluble fraction, according to Collin et al. (35). Casein fractions were expressed as the percentage of the total nitrogen (determined using the Kjeldahl method). In addition, characteristic peptides derived from the hydrolysis of β-casein in water-soluble extracts (γ_1_-CN, γ_3_-CN and γ_2_-CN) were analyzed by SDS-PAGE as previously described (21).

Short-chain carboxylic acids (acetic, propionic, butyric and caproic acids) were analyzed by gas chromatography after solvent extraction, as previously described (36). D- and L-lactate were analyzed in cheese water-soluble extracts by an enzymatic method using Boehringer kits (R-Biopharm, St. Didier au Mont d’Or, France).

### Analysis of Cheese Techno-Functional Properties

Four techno-functional properties were investigated on ripened cheeses. Cheese stretchability (3 replicates per sample) was assayed by a method involving vertical traction of the cheese melted at 82^°^C according to Richoux et al. (37). The length (mm) of strands of heated cheese was measured at the breaking point of the stretched strand. Oiling off was measured by a butyrometric method (37). Briefly, 3 g of ground cheese were melted at 65^°^C in a Van Gulik butyrometer placed in a water-bath for 20 min and the free oil was extracted by the addition of warm water (65^°^C) and centrifugation (350 *g* for 2 min). Results were expressed as g free oil per 100 g cheese or as g free oil per 100 g of fat, by dividing free oil by cheese fat content. Flowability was measured by a modified Schreiber test. Eight grams of ground cheese were molded and pressed to reconstitute a disc (4 cm diameter). This disc was put on a laser paper sheet and placed for 3 min in a domestic oven at 225^°^C. After cooling at room temperature, six radii of the melted cheese were measured and the area of the melted cheese evaluated. The melting index was calculated by dividing the area of the melted cheese with the area of the non-melted disc of cheese (37). The chewiness of the melted and partially defatted cheese obtained after free oil determination (around 45^°^C) was measured by the extrusion strength expressed in kgF using a previously developed device (38).

### Statistical Analyses

Two-way analyses of variance (ANOVA) were performed to determine whether the rate of casein degradation was affected by the NaCl level and the pH value, for each casein and LAB strain tested, using the R function *aov* (R version 3.6.2).

Regarding cheese data, two-way ANOVA were performed to determine whether the microbial, biochemical and techno-functional variables describing cheeses at each stage of ripening differed according to the NaCl content (three levels), the strain of *L. helveticus* used (three levels), and their interaction, using the R function *aov*. The means of three replicates were compared using the Tukey *post hoc* test from the R package *emmeans* (*p*-value < 0.05).

A Principal Component Analysis (PCA) was performed by using *FactoMineR* to illustrate the global biochemical and microbiological composition, and techno-functionalities of the 24 ripened cheeses manufactured (3 *L. helveticus* strain levels, 3 NaCl levels, 2–3 replicates).

## Results

### Lactic Starter Selection and Characterization

#### Verification of the Presence of Cell-Envelope Proteinase Genes in the Selected *Lactobacillus helveticus* Strains

The PCR results to check the presence or absence of each of the four CEP genes are shown on Supplementary Figure 1. All strains possessed the expected CEP gene(s). For CIRM-BIA 99, RO052, only the specific amplicon of *prtH3*, or *prtH4* was detected, respectively, while the amplicons of the four CEP genes were detected for CIRM-BIA103, with the amplicon size corresponding to those described by Broadbent et al. (23).

### Growth and Milk Acidification of Selected Lactic Starters

The maximum acidification rates and populations of *L. helveticus* strains varied over a large range (Table 2). Two strains, CIRM-BIA103 and CIRM-BIA99, reached high cell numbers (∼9 log_10_ CFU.mL^–1^) and acidified milk at a 3–4-fold greater rate compared to the slowest strain, RO052, which hardly grew during incubation. To verify that the latter strain was able to grow in milk, it was also inoculated at a low level of 4.9 log_10_ CFU.mL^–1^. After 40 h incubation, bacterial counts reached 6.7 log_10_ CFU.mL^–1^ and showed a low acidification rate (Table 2).

**TABLE 2:** Growth and acidification rates of three *Lactobacillus helveticus* strains possessing different CEP genes.

	Counts, log_10_ CFU.mL^–1^		Acidification rate, UpH.h^–1^ ^c^	
*L. helveticus* strains^a^	Inoculation	Stationary phase^b^	Mean	SEM
CIRM-BIA103	6.94	8.98	0.37	± 0.01
CIRM-BIA99	6.97	8.91	0.27	± 0.00
RO052	6.65	<6.3	0.09	± 0.00
RO052	4.90	6.7^d^	0.04	± 0.00

*^a^See Table 1.*

*^b^Reached after approximately 12 h.*

*^c^UpH, pH units; SEM, Standard Error of the Mean.*

*^d^After 40 h incubation.*

#### Cell-Envelope Proteinases Detection by Shaving of the Cell Surface of *Lactobacillus helveticus* Strains

To determine whether CEPs were produced, the three *L. helveticus* strains were subjected to shaving after growth in milk. Twelve and 37 proteins were identified for strains CIRM-BIA99 and CIRM-BIA103, respectively, including CEPs (Supplementary Table 1). The number of specific identified peptides of CEPs is presented in Supplementary Table 2. Peptides from the four CEPs PrtH, PrtH2, PrtH3, and PrtH4 were effectively detected for CIRM-BIA103, as expected. Concerning the other strains, only peptides from PrtH3 and PrtH4 were produced by CIRM-BIA99 and RO052, respectively, as expected (Supplementary Table 2).

### Capability of *Lactobacillus helveticus* Strains to Hydrolyze Purified α_s1_- and β-Caseins at Different pH and Salt Concentrations

**FIGURE 1:**
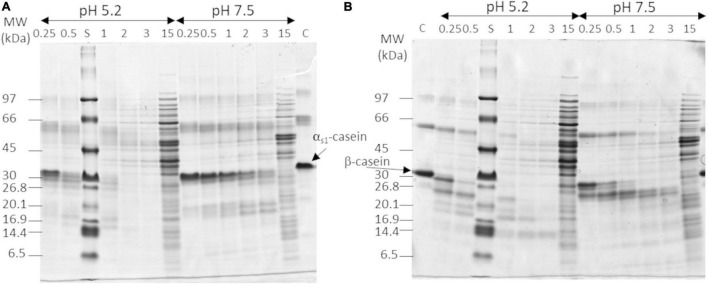
*In vitro* hydrolysis of **(A)** α_s1_-casein and **(B)** β-casein by *Lactobacillus helveticus* CIRM-BIA103 observed in Tris-tricine SDS-PAGE using 12–18% acrylamide gradient at NaCl 1.5% NaCl/moisture. Hydrolyzes were performed at pH 5.2 and 7.5 and samples were collected at six time points: 0.25, 0.5, 1 h, 2 h, 3 h, and 15 h. Molecular weight (MW) range of apparent molecular mass separation from 97 to 6.5 kDa are shown on the left side of each gel. Controls (C) are **(A)** α_s1_-casein in pH 7.5 Tris-buffer and **(B)** β-casein in pH 5.2 lactate buffer.

The proteolytic activity of the three *L. helveticus* strains was quantified on purified α_s1_- and β-caseins at two pH and six NaCl concentrations. Figure 1 shows the gels for *L. helveticus* CIRM-BIA103 at the median NaCl concentration (1.5%). The rate of casein hydrolysis was higher at pH 5.2 than at pH 7.5 regardless of the casein and the salt content for CIRM-BIA103 (Figure 1). The hydrolysis of α_s1_- and β-caseins generated a set of peptides, which migrated below 30 kDa with an increasing area during hydrolysis. Bands of molecular mass above 30 kDa, which corresponded to intracellular proteins released *via* the lysis of bacterial cells, appeared after 2 h of incubation in most of the conditions tested, and even after 30 min at the highest salt concentration tested, i.e., 4.5% NaCl (w/v) (Supplementary Figure 2). LAB lysis, which is commonly observed in LAB cultures and in cheese (25, 39), induced the release of peptides and amino acids in the medium (40, 41).

**FIGURE 2:**
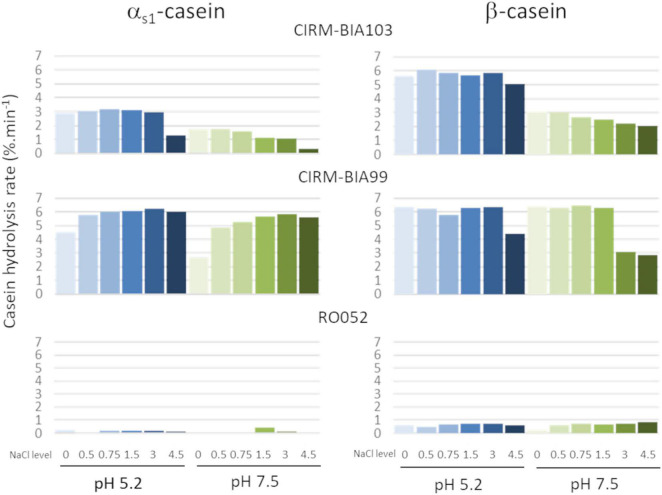
Rate of α_s1_- and β-caseins hydrolysis by three *Lactobacillus helveticus* strains possessing different CEP genes, at two pH (5.2, in blue and 7.5, in green) and six NaCl concentrations (0, 0.5, 0.75, 1.5, 3.0, and 4.5, from light color to dark color). Strains: *L. helveticus* CIRM-BIA103, CIRM-BIA99, and RO052.

The rate of casein hydrolysis by four strains was calculated from the decrease of intensity of casein bands in SDS PAGE at both pH and the six NaCl concentrations tested and is plotted in Figure 2. Huge differences were observed according to the strain. The most proteolytic strains were *L. helveticus* CIRM-BIA103 and CIRM-BIA99, with a hydrolysis rate of up to 5%.min^–1^. The latter hydrolyzed both α_s1_- and β-caseins, while the former preferentially hydrolyzed β-casein. *L. helveticus* RO052 weakly hydrolyzed β-casein and nearly not α_s1_-casein.

As for salt effect, the higher rates were observed at values below 1.5% NaCl, regardless of the strain, the casein, and the pH. Above this concentration, the proteolytic activity decreased in a variable manner according to the casein and the conditions. For example, a twofold lower rate of hydrolysis of β-casein was observed for *L. helveticus* CIRM-BIA99 at pH 7.5 and NaCl 3–4.5%, while the rate of hydrolysis of α_s1_-casein remained almost constant for this strain at all pHs and salt levels. In contrast, for *L. helveticus* CIRM-BIA103 the effect of high salt content on the rate of hydrolysis of α_s1_-casein was more pronounced compared to that of β-casein, with up to ninefold decrease at pH 7.5 and 4.5% NaCl (Figure 2).

Regarding the effect of pH, the hydrolysis rate was around 2 times higher at pH 5.2 compared to pH 7.5 for *L. helveticus* CIRM-BIA103 for both caseins, while it was only slightly higher for α_s1_-casein (+ 16%, *p* = 0.016) for *L. helveticus* CIRM-BIA99. No significant differences were observed for *L. helveticus* RO052 (Figure 2).

### Characteristics of Swiss-Type Cheeses Manufactured With Different *Lactobacillus helveticus* Starters and Salt Concentrations

#### Cheese Biochemical and Microbial Composition

Cheese microbial and biochemical composition over the ripening period are given in Table 3. for the nine types of cheeses: three *L. helveticus* strains (either CIRM-BIA103, CIRM-BIA99, or no *L. helveticus* strain), each at three NaCl concentration (regular salt concentration N1, i.e., 1.2% salt/moisture, lower (N0.7) and higher (N2.5) salt concentrations.

**TABLE 3:** Bacterial and biochemical composition of Swiss cheeses manufactured with different NaCl contents and *Lactobacillus helveticus* strains.

Cheeses			Bacterial counts, log10 CFU.mL^–1^			*pH*	Casein hydrolysis, % of total N	
Time of ripening	*Lactobacillus helveticus* strains	Salt level	*S. thermophilus*	*L. helveticus*	*P. freudenreichii*		β -casein	α_s1_-casein
D1	Lh103	N0.7	8.49^ab^	8.83^ab^	4.84^ab^	5.05^a^	28.05^ab^	46.13^a^
		N1	8.15^b^	8.40^b^	4.60^ab^	5.07^a^	29.37^a^	47.80^a^
		N2.5	8.47^ab^	8.74^ab^	5.00^a^	5.13^a^	28.98^ab^	45.24^a^
	Lh99	N0.7	8.37^ab^	9.12^ab^	4.77^ab^	5.03^a^	26.06^ab^	43.99^a^
		N1	8.32^ab^	9.07^ab^	4.82^ab^	5.07^a^	26.20^ab^	43.45^a^
		N2.5	8.49^ab^	9.16^a^	4.54^ab^	5.13^a^	25.47^ab^	43.82^a^
	NoLh	N0.7	8.47^ab^	NA	4.21^b^	5.12^a^	26.05^ab^	43.56^a^
		N1	8.98^a^	NA	4.51^ab^	5.14^a^	24.38^b^	43.31^a^
		N2.5	8.81^ab^	NA	4.44^ab^	5.04^a^	27.74^ab^	45.21^a^
D20	Lh103	N0.7	7.89^bc^	6.88^b^	5.00^a^	5.48^a^	24.34^ab^	36.41^ab^
		N1	7.68^c^	7.41^ab^	4.91^ab^	5.38^ab^	24.04^ab^	36.97^ab^
		N2.5	8.09^abc^	6.61^b^	5.01^a^	5.31^bc^	25.23^ab^	42.41^a^
	Lh99	N0.7	8.09^abc^	7.51^ab^	4.85^ab^	5.38^ab^	23.15^b^	32.54^a^
		N1	8.28^abc^	7.57^ab^	4.92^ab^	5.32^bc^	24.45^ab^	40.04^ab^
		N2.5	8.22^abc^	8.34^a^	4.82^abc^	5.34^bc^	27.79^a^	39.15^ab^
	NoLh	N0.7	8.46^ab^	NA	4.49^c^	5.35^bc^	22.96^ab^	39.80^ab^
		N1	8.58^a^	NA	4.62^bc^	5.32^bc^	23.43^ab^	40.57^ab^
		N2.5	8.73^a^	NA	4.70^abc^	5.24^c^	24.08^ab^	38.27^ab^
D34	Lh103	N0.7	6.37^de^	6.14^a^	6.16^ab^	5.34^a^	21.26^abc^	32.74^ab^
		N1	5.95^e^	5.83^a^	6.80^a^	5.43^a^	21.07^bc^	31.82^ab^
		N2.5	6.75^cd^	5.73^a^	6.46^a^	5.39^a^	22.71^abc^	28.83^ab^
	Lh99	N0.7	7.17^bc^	3.28^b^	6.73^a^	5.40^a^	20.51^c^	30.23^ab^
		N1	7.35^bc^	3.75^b^	6.06^ab^	ND	24.83^a^	30.85^ab^
		N2.5	7.64^ab^	5.72^a^	5.09^bc^	ND	24.34^ab^	33.71^a^
	NoLh	N0.7	8.40^a^	NA	5.11^bc^	5.39^a^	19.54^c^	25.15^b^
		N1	8.44^a^	NA	5.20^bc^	5.33^a^	19.99^c^	29.62^ab^
		N2.5	8.35^a^	NA	4.24^c^	5.19^b^	21.96^abc^	29.63^ab^
D48	Lh103	N0.7	5.13^e^	5.60^a^	8.55^ab^	5.49^a^	14.47^a^	21.34^bc^
		N1	4.97^e^	5.42^a^	8.82^a^	5.49^a^	12.40^a^	20.94^bc^
		N2.5	5.84^d^	5.94^a^	7.98^ab^	5.38^abc^	17.02^a^	23.08^bc^
	Lh99	N0.7	6.14^d^	3.11^b^	8.79^a^	5.45^a^	14.72^a^	23.78^bc^
		N1	6.44^cd^	3.48^b^	8.37^ab^	5.40^ab^	16.87^a^	19.95^c^
		N2.5	7.05^bc^	3.66^b^	7.41^bc^	5.41^ab^	18.83^a^	23.11^bc^
	NoLh	N0.7	7.97^a^	NA	6.69^c^	5.34^abc^	16.10^a^	27.84^abc^
		N1	7.74^ab^	NA	7.29^bc^	5.26^bc^	16.44^a^	29.44^ab^
		N2.5	7.97^a^	NA	4.00^d^	5.20^c^	17.34^a^	33.64^a^

*Values are means of three, or two for NoLh cheeses, replicates. ^a,b,c,d,e^ Means within the same column at each ripening stage with no common superscripts, significantly differ (P < 0.05).*

*Sample codes are as follows: D01, D20, D34, D48: ripening stage, in days. L. helveticus strains: Lh103 (CIRM-BIA103), Lh99 (CIRM-BIA99), or NoLh (without L. helveticus), followed by the NaCl content: N1 (regular salt concentration, i.e., 0.45 g NaCl/100 g cheese), N0.7 (N1x0.7), and N2.5 (N1x2.5).*

*NA, not applicable; ND, not determined.*

The technology of cheese making was successfully adapted so as (i) to keep constant the dry matter regardless of the salting level, and (ii) to reach the expected pH of 5.2 at day 1 by adding supplemental lactose in milk for the manufacture of the cheeses without *L. helveticus* strain. The targeted composition was reached, with DM = 60.2 ± 0.87%, fat = 28.5 ± 0.52%, FDM = 47.3 ± 0.62%, MNFS 55.6 ± 0.96% and protein (total nitrogen × 6.38) of 27.4 ± 0.52% at day 1. Significant but slight differences of these parameters were observed at day 1, with N2.5 cheeses containing slightly less DM (59.3% vs. 60.8% in regular cheeses). The composition of ripened cheese (D48) was similar, with DM = 61.4 ± 0.69%, fat = 29.0 ± 0.52%, FDM = 47.2 ± 0.67%, MNFS 54.3 ± 0.81% and nitrogen matter of 27.8 ± 0.46.

The pH at day 1 was 5.09 ± 0.05 and did not vary according to the *L. helveticus* strain and the NaCl concentration. It increased at a significantly higher value in cheeses with both *L. helveticus* strain compared to cheeses without *L. helveticus* strain (pH 5.46, 5.42, and 5.27, respectively) (Table 3).

The NaCl content in ripened cheeses was 1.14 ± 0.24, 0.45 ± 0.07, and 0.33 ± 0.04 g NaCl/100 g cheese in cheeses N2.5, N1 and N0.7, respectively, i.e., a change by factors of 2.55 and 0.72 in cheeses N2.5 and N0.7, respectively, compared to the regular cheese N1.

At the beginning of ripening, the density of lactic starters was ∼8.5–9.0 log_10_ CFU.g^–1^. The counts of *S. thermophilus* ranged from 8.4 log_10_ CFU.g^–1^ when it was associated with a *L. helveticus* strain, to 8.8 log_10_ CFU.g^–1^ in the absence of *L. helveticus*. *S. thermophilus* viable counts then decreased over the ripening period, with a markedly better survival in the absence of *L. helveticus* and in N2.5 cheeses. Similarly, both *L. helveticus* strains decreased in viability during the ripening, reaching 5.7 and 3.4 log_10_ CFU.g^–1^ for strains CIRM-BIA103 and CIRM-BIA99, respectively. This decrease was observed regardless of the NaCl content, but was attenuated in N2.5 cheeses (Table 3).

*P. freudenreichii* grew during the ripening of cheese at 24^°^C. Its growth was markedly impacted by both the presence of *L. helveticus* and the NaCl content, with around 200 times lower counts at the end of ripening in the absence of *L. helveticus* and 50 times lower counts, on average, at the highest NaCl content. Its growth did not significantly differ in N1 and N0.7 cheeses.

#### Cheese Proteolysis

**FIGURE 3:**
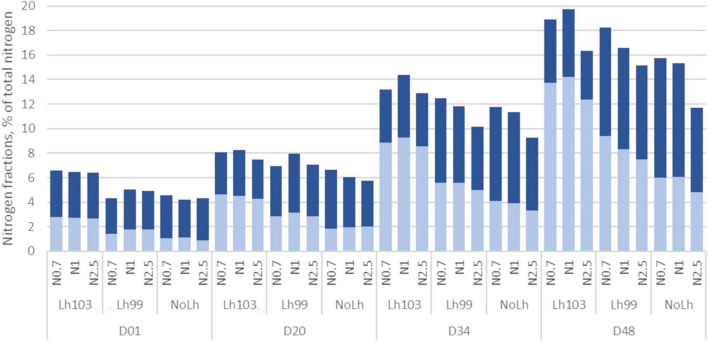
Changes in nitrogen fractions during the ripening of Swiss cheese manufactured with different *Lactobacillus helveticus* strains and NaCl content. Non-casein nitrogen (NCN, total bar) correspond to the pH4.6-soluble N, non-protein N (NPN, light blue) to the 12%TCA-soluble N. The difference “NCN minus NPN” is shown in dark blue. Values are means of three, or two for NoLh cheeses, biological replicates, and expressed as percentage of total nitrogen. Samples codes are as follows: D01, D20, D34, D48: ripening stage, in days. *L. helveticus* strain: Lh103 (CIRM-BIA103), Lh99 (CIRM-BIA99), or NoLh (without *L. helveticus*). NaCl content: N1 (regular salt concentration, i.e., 0.45 g NaCl/100 g cheese), N0.7 (N1 × 0.7), and N2.5 (N1 × 2.5).

The proteolysis of caseins was characterized over the ripening by the production of different N fractions: proteins and peptides soluble at pH 4.6 (NCN) and smaller peptides (TCA 12%-soluble N, referred to as NPN) (Figure 3). In addition, the hydrolysis of α_s1_- and β-caseins were followed by electrophoresis at the end of the ripening (Table 3).

The NCN fraction increased over the ripening from 5.3% of TN at day 1, to reach 7.3, 12.1, and 16.7% of TN at days 20, 34, and 48, respectively. Similarly, the NPN fraction increased from 1.9% of TN at day 1 to 3.3, 8.9, and 9.6% of total N at days 20, 34, and 48, respectively. As a consequence, the highest molecular mass peptides (NCN minus NPN) decreased in proportion of the NCN fraction, indicating the accumulation of smaller ones from the hydrolysis of the largest peptides (Figure 3).

Proteolysis was impacted by both *L. helveticus* strain and NaCl concentration, as illustrated in Figure 3 for the NCN and NPN fractions, expressed as percentage of TN. Proteolysis was significantly higher in the presence of *L. helveticus* CIRM-BIA103 over the whole ripening period, followed by CIRM-BIA99, while NoLh cheeses contained the lowest proteolysis level. Regarding the NaCl effect, proteolysis was similar in the cheeses with a regular (N1) and a low (N0.7) salt content, and lower at a high salt content. For example, the NCN fraction at day 48 was 1.7 times greater in cheese containing *L. helveticus* CIRM-BIA103 and a regular salt content, compared to the cheese without *L. helveticus* and with the highest NaCl content, while the NPN fraction was 2.9 times greater between these the same two extreme cheeses.

Regarding casein hydrolysis, the degradation of α_s1_- and β-caseins at day 1 (i.e., before salting) did not differ by more than 12% between cheeses as function of *L. helveticus* strains (Table 3). At day 48, no significant differences in β-casein hydrolysis and γ_2_-CN were observed between strains (*p* > 0.05), while α_s1_-casein hydrolysis was 24% higher in the presence of *L. helveticus* than in NoLh cheeses and γ_1+3_-CN 33% higher in the presence of *L. helveticus* CIRM-BIA103 than in other cheeses (Table 3 for α_s1_- and β-caseins, results not shown for γ_2_-CN and γ_1+3_-CN). The NaCl level did not significantly affect α_s1_- and β-casein hydrolysis (Table 3), and the abundance of the derived peptides (results not shown), despite a tendency (−12%, *p* = 0.11) of slowdown of β-casein hydrolysis at the highest salt level.

#### Cheese Techno-Functional Properties

Four techno-functional properties of cheeses were also determined at the end of ripening (Table 4). Cheese stretchability reached a mean value of 536 mm, with high variations depending on the *L. helveticus* strain. It was 2.0 and 1.4 times greater in the presence of *L. helveticus* CIRM-BIA103 and CIRM-BIA99 cheeses, respectively, compared to the cheeses that did not contain *L. helveticus*, regardless of the salt content. The extrusion value significantly reached a mean value of 8.3 kgF. It significantly varied with both factors (*p* < 0.05). It was 31% lower, on average, in the absence of *L. helveticus* strain, and 17% lower at the lowest salt content compared to regular salt cheese. The oiling off values significantly varied with both factors (*p* < 0.05). It was 17 and 11% higher in cheeses which contained *L. helveticus* CIRM-BIA103 and CIRM-BIA99, respectively, compared to NoLh cheeses, and 4% lower at the lowest NaCl content (*p* < 0.05). Flowability did not significantly vary according to the cheese type.

**TABLE 4:** Techno-functional properties of Swiss cheeses manufactured using different *Lactobacillus helveticus* strains and NaCl concentrations.

Functional properties	*L helveticus* CIRM-BIA103			*L helveticus* CIRM-BIA99			No *L helveticus*		
	N0.7	N1	N2.5	N0.7	N1	N2.5	N0.7	N1	N2.5
Stretchability (mm)	833^ a^	642^ab^	625^abc^	397^cde^	471^bcde^	607^abcd^	357^de^	304^e^	402^bcde^
Flowability	2.21^a^	2.41^a^	2.77^a^	2.15^a^	2.23^a^	2.42^a^	2.22^a^	2.06^a^	2.27^a^
Fat oiling off (%)	12.6^cd^	14.2^a^	13.8^ab^	12.0^de^	13.2^cd^	13.3^b^	12.4^de^	11.3^ef^	11^f^
Extrusion (kgF)	7.7^bc^	10.6^a^	10.2^ ab^	7.9^bc^	8.8^ab^	10.1^ab^	5.2^c^	5.7^c^	8.3^abc^

*^a,b,c,d,e,f^Means within a row with no common superscripts significantly differ (P < 0.05).*

*The manufacturing conditions were adapted to reach a similar composition at day 1. Values are means of three (or two for NoLh cheeses) biological replicates at 48 days of ripening. NaCl content: N1 (regular salt concentration, i.e., 0.45 g NaCl/100 g cheese), N0.7 (N1x0.7), and N2.5 (N1x2.5).*

### Global Characteristics of Ripened Cheeses

**FIGURE 4:**
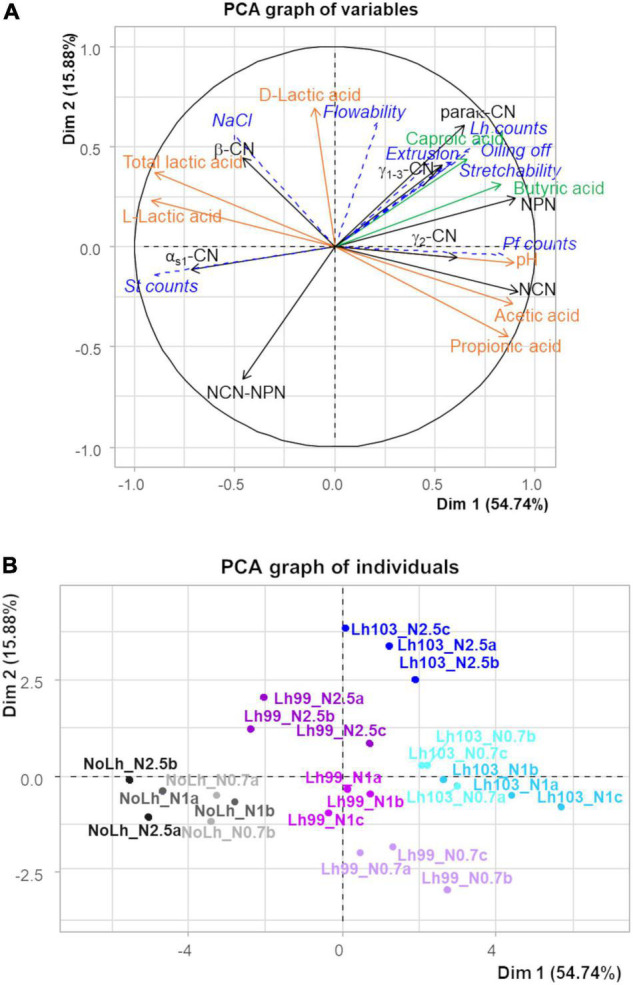
Variable map **(A)** and individual factor map **(B)** of the two first principal components of principal component analysis of 16 biochemical variables, which characterize proteolysis (in black), lipolysis (in green), and lactic and propionic acid fermentation (in orange) in Swiss cheeses manufactured with different *Lactobacillus helveticus* strains and NaCl contents, at the end of the ripening (48 days). A set of 8 other variables that describe microbial counts, NaCl content, and techno-functional properties are projected as supplementary variables (in italics and blue dashed lines). Samples codes are as follows: *L. helveticus* strain: Lh103 (CIRM-BIA103), Lh99 (CIRM-BIA99), or NoLh (without *L. helveticus*), followed by the NaCl content: N1 (regular salt concentration, i.e., 0.45 g NaCl/100 g cheese), N0.7 (N1 × 0.7), and N2.5 (N1 × 2.5). Triplicates, coded a, b, and c, were performed for each salt concentration and *L. helveticus* strain used, except NoLh cheeses manufactured in duplicate, coded a and b.

To summarize and illustrate all the results, a PCA was performed by using data at the end of ripening for all cheese types. The variables that characterized proteolysis, lipolysis, and fermentation of lactic acid were used as active variables, while microbiological variables, gross composition, and techno-functional properties were projected as supplementary variables (Figure 4). The first two dimensions explained 70.6% of the total variability. The nine cheese types formed seven groups: cheeses with the strain *L. helveticus* CIRM-BIA99 at each salt level (shown in three shades of purple), cheeses with the strain *L. helveticus* CIRM-BIA103 at the highest salt level (in dark blue), and cheeses without *L. helveticus* at the highest salt level (in black) were grouped individually, while cheeses with *L. helveticus* CIRM-BIA103 at low and regular salt content (two shades of light blue) were grouped together, as were also cheeses without *L. helveticus* at low and regular salt content (two shades of gray).

The first axis accounted for 54.7% of the total variability. It was positively related to the main biochemical events: proteolysis (NPN, NCN, γ_1+3_-CN derived from the hydrolysis of β-casein), lipolysis (caproic and butyric acids derived from fat hydrolysis), and conversion of lactic acid to propionic and acetic acids, and, conversely, negatively correlated to α_s1_-casein and total lactic acid concentration. This axis was positively associated with the cheeses containing the most proteolytic *L. helveticus* strain, CIRM-BIA103, and negatively associated with the cheese that did not contain *L. helveticus*. Regarding supplementary variables, high counts of propionibacteria (Pf_counts) and three functional properties (stretchability, extrusion, and oiling off) were correlated with proteolysis, in particular NPN fraction, and associated with cheeses with *L. helveticus*, in particular with strain CIRM-BIA103 (Lh103). In contrast, high counts of *S. thermophilus* (St_counts) were associated with cheeses that did not contain *L. helveticus* (NoLh cheeses).

The second axis, which accounted for 15.9% of the total variability, was positively associated with high concentrations of D-lactic acid associated with the cheeses containing both a *L. helveticus* strain and a high NaCl content, and negatively associated with high content of the fraction NCN-NPN, i.e., high molecular mass peptides.

## Discussion

### Salt Reduction in Food

In the context of salt reduction in diet, the European TeRiFiQ project addressed several food categories, both fermented or not (7). The case of fermented foods is peculiar, because it is a living product that continuously evolves throughout process and storage. Fermented foods actually contain a more or less complex microbiota, the metabolism of which converts cheese components into a series of desirable compounds *via* carbohydrate fermentation, proteolysis, and lipolysis, under favorable conditions of process (42). The balance between the desirable and undesirable (spoiling or pathogenic) micro-organism of fermented foods is greatly impacted by the physicochemical conditions, including the salt concentration. The challenge of salt reduction is to keep the fermented food quality.

### Strategy Chosen: Bacterial Strains With Different Proteolytic Systems Tested *in vitro* and in Cheese Experiments at Different Salt Contents

The cheeses studied here are internally bacterial-ripened hard cheeses, which are manufactured with thermophilic lactic acid bacteria (LAB) as starters and most generally associate the *S. thermophilus* and *L. helveticus* species. As proteolysis is the most important biochemical event during the ripening of most cheese varieties and that it markedly depends on the strains of starters used in manufacture (43), we chose to select different *L. helveticus* strains to investigate the effect of salt concentration on their proteolytic activity. LAB possess cell-envelope proteinase(s) (CEP) that are required to support their growth in milk, by hydrolyzing caseins, the main milk proteins, into peptides and free amino acids that can be used for their nitrogen requirements (42). *L. helveticus* possesses one CEP, PrtH, and up to four proteinases, depending on the strain (20). *L. helveticus* CEPs have been characterized for their ability to differently hydrolyze β- and α_s1_-caseins *in vitro* (21), but little was known on their activity under cheese conditions, and the resulting consequences on the overall quality of cheese.

In this study, after validating that the selected *L. helveticus* strains effectively possessed the expected CEP genes, we demonstrated that they actually expressed all these genes during their growth in milk (Supplementary Figure 1). We then studied the proteolytic activity of these strains *in vitro* under different salt and pH conditions, before comparing the impact of selected strains in experimental Swiss-type cheeses manufactured with regular, lower, and higher NaCl concentrations. These salt levels correspond to a 30% reduction, in accordance to the WHO recommendations (2), and to a 2.5-fold higher salt content to mimic the concentration near the cheese rind or the one present in other hard cheese varieties (4).

### Impact of Salt Change on Proteolysis by Lactic Acid Bacteria

The strategy chosen was to investigate the proteolytic activity of the selected *L. helveticus* strains on the two main caseins, α_s1_- and β-caseins, at six salt concentrations representative of the ones of various hard cheeses (4) and at two pH, the pH of hard cheese before ripening and a neutral pH as usually done under *in vitro* conditions (19). Our result showed *in vitro* a similar or twofold higher casein hydrolysis rate at pH 5.2 compared to pH 7.5, for the two higher proteolytic *L. helveticus* strains, CIRM-BIA99 and CIRM-BIA103, respectively. Regarding salt effect, casein hydrolysis only decreased at the highest salt concentrations (3.0 and 4.5% NaCl) (Figures 1, 2). The impact of pH and/or salt on CEP activity had been mainly studied for PrtP, the CEP of *Lactococcus lactis*, on a specific peptide that result from hydrolysis of α_s1_-caseins by chymosin, α_s1_-casein (1–23) peptide. For example, differences in specificity of hydrolysis by PrtP were observed, leading to fewer bounds of α_s1_-casein (1–23) peptide hydrolyzed at pH 5.2 and in the presence of 4% salt than at pH 6.5 in the absence of salt (44–46), in contrast to the results observed in our study on *L. helveticus* CEPs. A higher proteolytic activity on α_s1_- and β-caseins at pH 5.2 was also observed for chymosin, used as coagulant in cheese (44). These results stress the importance of studying proteolytic activity under cheese physicochemical conditions to select strains in a relevant way.

In cheeses, a 30% salt-reduction did not affect proteolysis, while only a slight decrease (−14%) of the non-protein fraction was observed in the 2.5-fold higher salted cheeses containing 1.14 g/100 g cheese, i.e., 2.9% salt in moisture (Figure 3 and Table 3), in agreement with the results observed *in vitro*. Similarly, in semi-hard cheeses such as Prato, Trappist and Reblochon-type cheeses, salt reduction did not significantly affect their biochemical composition and sensory characteristics (7, 9, 47). However, the impact of salt reduction on the final cheese characteristics greatly depends on the cheese type. For instance, a 20%-NaCl reduction in a soft (Camembert-type) cheese led to a 29% increase of proteolysis evaluated by the non-protein fraction and a 19% increase of lipolysis (9). Moreover, some defects occurred in 30%-reduced salt Trappist cheese manufactured in Winter, due to butyric acid fermentation caused by the *Clostridium* spoilage (7). Similarly, in a soft cheese with smear rind, a 30% salt reduction led to the undesirable and unresolved growth of white molds (7).

In our study, cheese proteolysis was markedly more impacted by the *L. helveticus* strain used than by the changes in salt concentration. The cheeses manufactured with the two selected strains of *L. helveticus* showed 1.5–2.4 more proteolysis, estimated by the non-protein fraction, compared to the cheeses without *L. helveticus*. This can be due to the large differences in number of CEPs in LAB, as shown in the present study for the three *L. helveticus* strains characterized and for other LAB (19). The highest activity was observed for the *L. helveticus* strains that expressed the CEP PrtH3, either alone as for CIRM-BIA99 and in addition to other CEPs as for CIRM-BIA103, while a very low activity was detected for *L. helveticus* RO052 that harbors only the CEP PrtH4. Therefore, the choice of LAB strains used as starter in cheese offers opportunities to counteract changes in salt concentration or other changes in physicochemical conditions.

Besides the CEP they possess, other traits of LAB can influence cheese proteolysis, including (i) the level of expressions of CEP genes (23), which modulates the amount of CEP anchored at the cell surface, and (ii) the capacities of lysis during cheese ripening (39). In our study, LAB lysis was not evaluated but the concomitant dramatic decrease in *L. helveticus* viable counts in 8-day cheeses and increase in proteolysis suggest that *L. helveticus* cells may have lysed in cheeses, as frequently observed in cheese (48) and observed *in vitro.* The lysis observed *in vitro* was higher for *L. helveticus* CIRM-BIA103 at salt contents above 0.75% from visualization of intracellular proteins on SDS PAGE gels (Supplementary Figure 2). Similarly, another *L. helveticus* strain having the four CEPs, ITGLH1, also showed early lysis in cheese (17).

### Impact of Salt Reduction on Overall Cheese Properties

Propionibacteria are used as a ripening cultures in Swiss-type cheese, where they are involved in flavor development due to their lipolytic and amino acid-converting activities (48, 49). Some strains are also associated with probiotics properties (50), e.g., immunomodulation in the intestinal tract. In the present study, propionibacteria growth was not affected by salt reduction, neither the concentration of propionic and acetic acids, which results from their activity. In contrast, we observed a marked effect of salt increase on propionibacteria growth and metabolic activity (Table 3 and Figure 4), in agreement with previous studies (51, 52). Here again, salt resistance is highly strain-dependent and the choice of the most adapted strains can be done for the ripening of high salted cheeses, as previously successfully done (53). Moreover, the growth of propionibacteria can be favored by *L. helveticus* strains, which provide peptides and free amino acids (54).

Regarding cheese techno-functional properties, two of the four properties evaluated, flowability, and stretchability, were not affected by salt reduction, while fat oiling off and extrusion were slightly lower (Table 4). In contrast, at the upper salt level, a tendency of higher flowability and extrusion of cheeses was observed (+ ∼10%, not significant difference). Moreover, the concentration of free amino acids and peptides, which are aroma precursors, and of short-chain fatty acids (acetic, propionic, butyric, and caproic acids), which are important flavor compounds in Swiss-type cheese, did not change with a 30% salt reduction, suggesting that the perception of flavor may be maintained, as shown in model cheeses (55). However, since no sensory evaluation was performed, we cannot exclude that the decrease of NaCl concentration *per se* was perceived. Generally, the other cheeses studied in the TeRiFiQ European project with a 30% salt reduction were evaluated as “slightly different from the original cheese” but remained “very acceptable by the consumers” (7).

## Conclusion

This study showed that reducing NaCl level by 30% is technically feasible without modifying cheeses characteristics of Swiss-type cheese. Moreover, it suggests that some of the changes induced by modifying salt concentration in Swiss-type and some other hard cheeses could be at least partially compensated by the choice of relevant starters used in cheese manufacture. These conclusions cannot be generalized to all cheese varieties, because the impact of salt reduction highly depends on the cheese type.

## Data Availability Statement

The datasets presented in this study can be found in online repositories. The names of the repository/repositories and accession number(s) can be found below: https://doi.org/10.15454/2KFMGC, Data INRAE.

## Author Contributions

XL and VG performed the experimental study. RR performed the cheese experiments and associated analyses. JJ and VB-B performed the MS analyses. MG, AT, and VG supervised the whole work. XL wrote the manuscript draft. AT and VG revised the manuscript. All authors approved the submitted version.

## Conflict of Interest

The authors declare that the research was conducted in the absence of any commercial or financial relationships that could be construed as a potential conflict of interest.

## Publisher’s Note

All claims expressed in this article are solely those of the authors and do not necessarily represent those of their affiliated organizations, or those of the publisher, the editors and the reviewers. Any product that may be evaluated in this article, or claim that may be made by its manufacturer, is not guaranteed or endorsed by the publisher.
