# Supplementary material for: Little Impact of NaCl Reduction in Swiss-Type Cheese
Source: Front Nutr. 2022 Jun 16;9:888179. doi: 10.3389/fnut.2022.888179 (PMC9243640; doi:10.3389/fnut.2022.888179)

**Supplemental Table S1:** Proteins identified after cell surface shaving of *Lactobacillus helveticus* strains CIRM-BIA103 (Lh103) and CIRM-BIA99 (Lh99), cell-envelope proteinases (CEP) are shown in bold

| Strain | Prot Id ^a^ | Description | MW | Peptide Nr ^b^ | Location ^c^ | Clues to predict location |
| --- | --- | --- | --- | --- | --- | --- |
| Lh103 | U6FFS9 | LHCIRMBIA103_02073 **Lactocepin H3 proteinase** | 178.70 | 23 | CS | Signal peptide |
|  | U6FJC5 | LHCIRMBIA103_02165 **Lactocepin H4 proteinase** | 174.90 | 5 | CS | Signal peptide |
|  | C5J2C0 | Tuf (fragment) | 29.50 | 11 | CS and Cyto | Henderson and Martin, 2011 ^d^ |
|  | A8YUV4 | Enolase | 46.60 | 11 | CS and Cyto | Henderson and Martin, 2011 |
|  | A8YV23 | Pyruvate kinase | 63.00 | 8 | CS and Cyto | Henderson and Martin, 2011 |
|  | A8YWA2 | Putative uncharacterized protein | 35.80 | 6 | CS | Signal peptide |
|  | A4ZGV7 | Fructose-1,6-biphosphate aldolase | 33.30 | 5 | CS and Cyto | Henderson and Martin, 2011 |
|  | C2EQS6 | L-lactate dehydrogenase | 35.10 | 5 | Cyto | predicted |
|  | O32765 | L-lactate dehydrogenase | 35.00 | 5 | Cyto | predicted |
|  | U6FHP9 | LHCIRMBIA103_00764 **CEP H2** | 192.30 | 5 | CS | Signal peptide |
|  | C9M4T8 | Putative uncharacterized protein (Fragment) | 69.50 | 5 | CS | Signal peptide |
|  | A4ZGY6 | 60 kDa chaperonin (groL) | 57.50 | 4 | CS and Cyto | Henderson and Martin, 2011 |
|  | U6FGX9 | LHCIRMBIA103_00753 **CEP H** | 207.50 | 4 | CS | Signal peptide |
|  | A8YUS3 | Trigger factor (tig) | 50.40 | 4 | CS and Cyto | Henderson and Martin, 2011 |
|  | A8YXK3 | Elongation factor G | 76.70 | 4 | Cyto | predicted |
|  | A8YWB1 | Penicillin-binding protein | 73.90 | 4 | CS | predicted |
|  | A4ZH15 | Phosphoribosylformylglycinamidine synthase 2 (purL) | 81.70 | 3 | Cyto | Swiss prot |
|  | A8YVQ3 | Chaperone protein DnaK | 65.60 | 2 | CS and Cyto | Henderson and Martin, 2011 |
|  | A8YXM2 | 30S ribosomal protein S5 (rpsE) | 17.90 | 2 | Cyto | predicted |
|  | A8YXL9 | 30S ribosomal protein S8 (rpsH) | 14.50 | 2 | Cyto | predicted |
|  | C9M3P2 | PTS family porter, phosphocarrier protein HPR (ptsH) | 9.30 | 3 | CS | predicted |
|  | A8YTF2 | 50S ribosomal protein L7/L12 (rplL) | 12.40 | 3 | Cyto | predicted |
|  | A8YWA1 | UDP-galactose 4-epimerase | 36.30 | 3 | Cyto | predicted |
|  | A8YUE3 | Phosphoglycerate kinase (pgk) | 42.70 | 3 | Cyto | predicted |
|  | A8YX23 | 2,3-bisphosphoglycerate-dependent phosphoglycerate mutase (gpmA) | 26.50 | 3 | Cyto | predicted |
|  | C9LZC9 | Oligopeptide ABC superfamily (oppA4) | 65.20 | 3 | CS | predicted |
|  | A8YW88 | Phosphoribosylaminoimidazole-succinocarboxamide synthase (purC) | 27.30 | 2 | Cyto | predicted |
|  | A8YUE4 | Triosephosphate isomerase | 27.50 | 2 | Cyto | predicted |
|  | A8YXM0 | 50S ribosomal protein L6 | 19.10 | 2 | Cyto | predicted |
|  | A8YVR9 | 30S ribosomal protein S2 (rpsB) | 28.70 | 2 | Cyto | predicted |
|  | A8YVR8 | Elongation factor Ts | 37.60 | 2 | Cyto | predicted |
|  | A8YW55 | Putative alkylphosphonate ABC transporter | 34.10 | 2 | CS | predicted |
|  | C9M349 | Xylulose-5-phosphate phosphoketolase (xpkA) | 91.20 | 2 | Cyto | predicted |
|  | A8YV36 | DNA-binding protein II HB | 9.70 | 2 | Cyto | predicted |
|  | A8YTF1 | 50S ribosomal protein L10 (rplJ) | 18.60 | 2 | Cyto | predicted |
|  | A4ZH30 | ATP-dependent Clp protease ATP-binding subunit(clpE2) | 79.20 | 2 | Cyto | predicted |
|  | A8YV34 | 30S ribosomal protein S1 | 44.20 | 2 | Cyto | predicted |
| Lh99 |  | **PrtH3** | 177.30 | 20 | CS | Signal peptide |
|  | A8YW31 | Putative uncharacterized protein | 68.30 | 5 | CS | Signal peptide |
|  | A8YWA2 | Putative uncharacterized protein | 35.80 | 3 | CS | Signal peptide |
|  | A8YUS2 | Elongation factor Tu (tuf) | 43.40 | 4 | CS and Cyto | Henderson and Martin, 2011 |
|  | A8YUE0 | Putative uncharacterized protein | 61.00 | 3 | CS | Signal peptide |
|  | C9LZC9 | Oligopeptide ABC superfamily | 65.20 | 3 | CS | predicted |
|  | P38059 | S-layer protein (slpH) | 46.60 | 3 | CS | predicted |
|  | A8YXB3 | Putative uncharacterized protein | 36.60 | 3 | CS | Signal peptide |
|  | A8YW55 | Putative alkylphosphonate ABC transporter | 34.10 | 3 | CS | predicted |
|  | A8YV23 | Pyruvate kinase | 63.00 | 3 | CS and Cyto | Henderson and Martin, 2011 |
|  | A7GMI4 | reversed SCP-like extracellular (Bcer98) | 31.40 | 2 | CS | predicted |
|  | A4ZGZ4 | D-alanyl-D-alanine carboxypeptidase | 46.60 | 2 | CS | predicted |
|  | A8YXK4 | 30S ribosomal protein S10 (rpsJ) | 11.40 | 2 | Cyto | predicted |

^a^ Prot Id: Identification sequence from the following databases TrEMBL or Swiss prot (in italic)

^b^ Number of identified peptides par identified protein

^c^ Locations of proteins: cell surface (CS) or cytoplasm (Cyto), predicted either by Uniprot data bank or by similarity to subcellular located proteins according to Camacho et al., 2009, BLAST plus: architecture and applications. BMC Bioinformatics. 10:421, doi:10.1186/1471-2105-10-421.

^d^ Henderson B, Martin A: Bacterial virulence in the moonlight: Multitasking bacterial moonlighting proteins are virulence determinants in infectious

disease. Infect Immun 2011, 79:3476–3491.

**Supplemental Table S2**: Identified peptides of cell-envelope proteinases (CEPs) after cell surface shaving of three *Lactobacillus helveticus* strains after hydrolysis by trypsine

| CEPs | Identified peptides | | |  | Peptide detected (D) in *L. helveticus* | | |
| --- | --- | --- | --- | --- | --- | --- | --- |
|  | Start | End | Sequences^a^ |  | CIRM-BIA103 | CIRM-BIA99 | RO052^b^ |
| PrtH |  |  |  |  |  |  |  |
|  | 335 | 357 | NMSLGSVSGEQTEDDPEVAAVER |  | D | - | - |
|  | 340 | 357 | SVSGEQTEDDPEVAAVER |  | D | - | - |
|  | 429 | 445 | TTIAGPEATQLSEGTDR |  | D | - | - |
|  | 938 | 954 | DDAPSWDGTYFDQQANK |  | D | - | - |
| PrtH2 |  |  |  |  | - | - | - |
|  | 65 | 81 | DAQAPSDDKQPDPVVQK* |  | D | - | - |
|  | 674 | 692 | ENVFDQNANDPKPDIQGNR |  | D | - | - |
|  | 693 | 701 | LVNEDNYPR |  | D | - | - |
|  | 783 | 810 | SYHSDGDGATVDLDNGATNSDVFDWDGK |  | D | - | - |
|  | 906 | 914 | NSGFDDANK |  | D | - | - |
| PrtH3 |  |  |  |  | - | - | - |
|  | 122 | 137 | DANGVELPANNQDHVK* |  | D | D | - |
|  | 157 | 167 | VIDSGIDVDHK |  | D | D | - |
|  | 158 | 167 | IDSGIDVDHK |  | D | - | - |
|  | 285 | 301 | SLGGGVSAADLNNADQR |  | D | D | - |
|  | 286 | 301 | LGGGVSAADLNNADQR |  | D | - | - |
|  | 332 | 344 | ITDLDDYEPGGER |  | D | D | - |
|  | 469 | 477 | GFDTPVSPR |  | - | D | - |
|  | 529 | 543 | AYDFDDMGGGFTEVR |  | D | D | - |
|  | 534 | 543 | DMGGGFTEVR |  | - | D | - |
|  | 565 | 574 | SVELAPNETK |  | D | D | - |
|  | 615 | 647 | GDMTSENVFDQNANDAAPDIQGNHLINEDNYPR |  | D | - | - |
|  | 627 | 647 | ANDAAPDIQGNHLINEDNYPR |  | - | D | - |
|  | 636 | 647 | GNHLINEDNYPR |  | - | D | - |
|  | 719 | 728 | VLSDNHGPEK |  | - | D | - |
|  | 742 | 755 | STVNNSDTLEWDGK |  | D | - | - |
|  | 792 | 808 | DTPVIIDTTAPVLNNVK |  | D | - | - |
|  | 867 | 882 | VLTPEEQAALTSAANK |  | D | - | - |
|  | 915 | 932 | AINGLPFNENSDDYNVGR |  | D | D | - |
|  | 916 | 932 | INGLPFNENSDDYNVGR |  | D | - | - |
|  | 921 | 932 | FNENSDDYNVGR |  | D | - | - |
|  | 922 | 932 | NENSDDYNVGR |  | D | D | - |
|  | 995 | 1004 | AWQHVDGEER |  | - | D | - |
|  | 1079 | 1091 | GWVEIDGPTFNAK |  | - | D | - |
|  | 1080 | 1091 | WVEIDGPTFNAK |  | D | - | - |
|  | 1198 | 1219 | FLADSPYEVDPENQADIHDNGK |  | D | - | - |
|  | 1200 | 1219 | ADSPYEVDPENQADIHDNGK |  | - | D | - |
|  | 1335 | 1350 | FDQEEQLDDQNGQPTT |  | D | - | - |
|  | 1387 | 1400 | KDDQADVKPAEGQK |  | D | D | - |
|  | 1388 | 1400 | DDQADVKPAEGQK |  | D | D | - |
|  | 1414 | 1435 | EDPESGQTTENAQSTESQEQNK |  | D | D | - |
|  | 1452 | 1467 | ENHGAGESTIESNQEK |  |  | D | - |
| PrtH4 |  |  |  |  |  |  |  |
|  | 162 | 173 | GNVQSAWDQGYR |  | - | - | D |
|  | 491 | 506 | AKPQTQLGYTTPVSPR |  |  | - | D |
|  | 559 | 572 | YTFDDYGGGYTEQR |  | D | - | - |
|  | 776 | 786 | DNPDALEWDGK |  | D | - | - |
|  | 1110 | 1122 | GWTEVDGPSFNDK |  | D | - | - |
|  | 1187 | 1205 | TDPNPDIHFDYMNDNDTTR |  | D | - | - |

a, Peptides belonging to the pro-peptide domains, identified for PrtH2 and PrtH3, are marked with an asterisk

b, Only two peptides of PrtH4 were identified in supernatant sample after 1 h of shaving by trypsin on RO052 cells surface and none after the second step of 20 h of hydrolysis (cf M&M)

**Figure S1:** Detection of the CEP genes *prtH*, *prtH2*, *prtH3* and *prtH4* by PCR amplification from genomic DNA of three *Lactobacillus helveticus* strains: CIRM-BIA103, CIRM-BIA99 and RO052 using the primers designed by Broadbent et al. (23) to specifically amplify fragments of each of the four CEP genes encountered in *L. helveticus* CIRM-BIA103. Amplicon size: *prtH*: 1.332 kb, *prtH2*: 4.042 kb, *prtH3*: 0.357 kb, and *prtH4*: 3.386 kb. C correspond to negative control of amplification by using RNA free water.


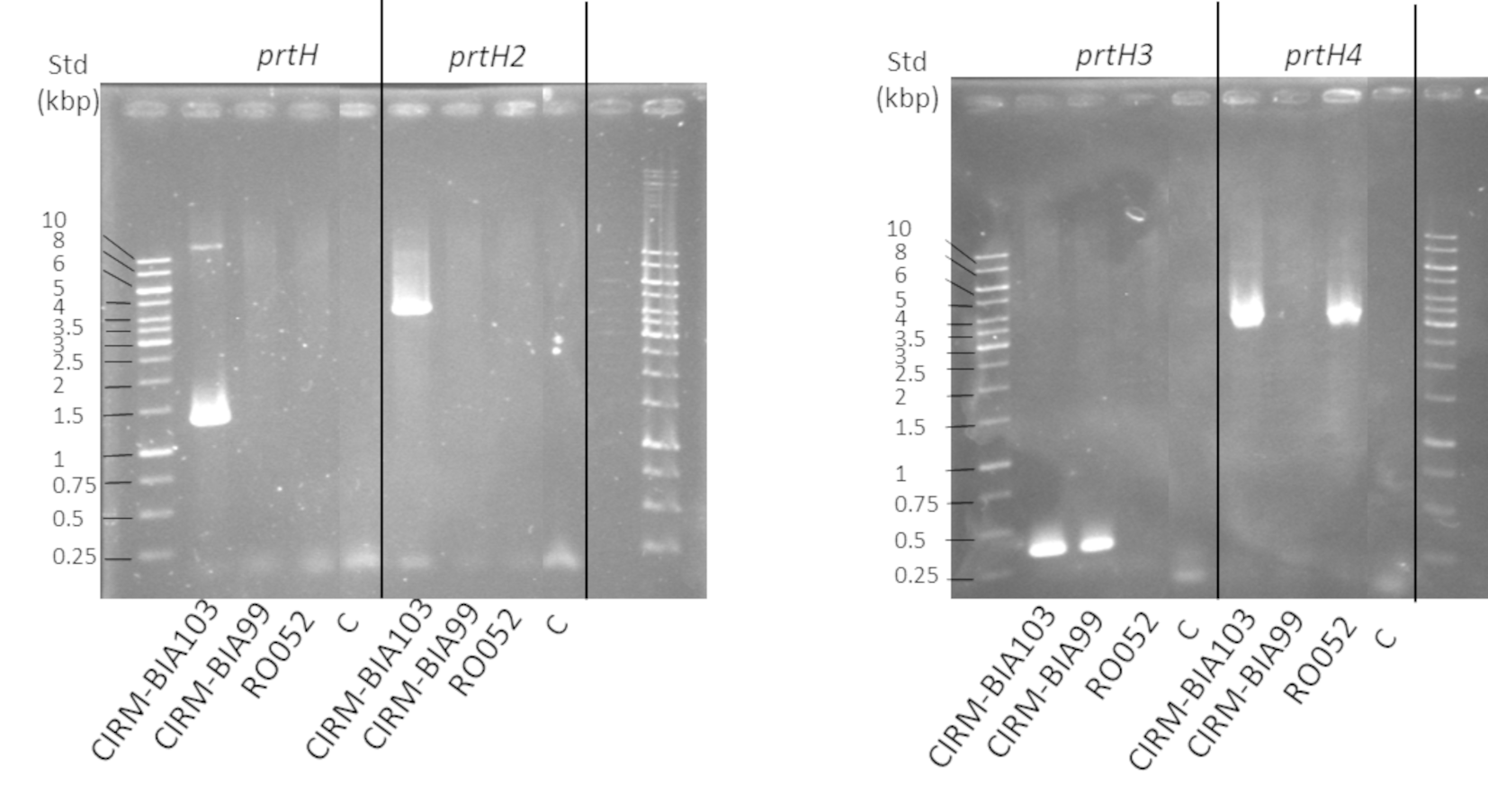


**Figure S2:** *In vitro* hydrolysis of the α_s1_- (Fig 2A, C and E) and β- (Fig 2B, D and F) caseins by three *Lactobacillus helveticus* strains (CIRM-BIA103, CIRM-BIA99 and RO052) observed in Tris-tricine SDS-PAGE using 12-18% acrylamide gradient at six NaCl concentrations (0% 0.5%, 0.75%, 1.5%, 3.0 and 4.5). Hydrolyses were performed at pH 5.2 in lactate buffer and 7.5 in Tris HCl-buffer. Samples were collected at six time points: 0.25, 0.5, 1 h, 2 h, 3 h, and 15 h. Molecular weight (MW) range of apparent molecular mass separation from 97 kDa to 6.5 kDa are shown on the left side of each gel and the corresponding lane labelled with a S. Controls (C) are α_s1_-casein or β-casein substrate.


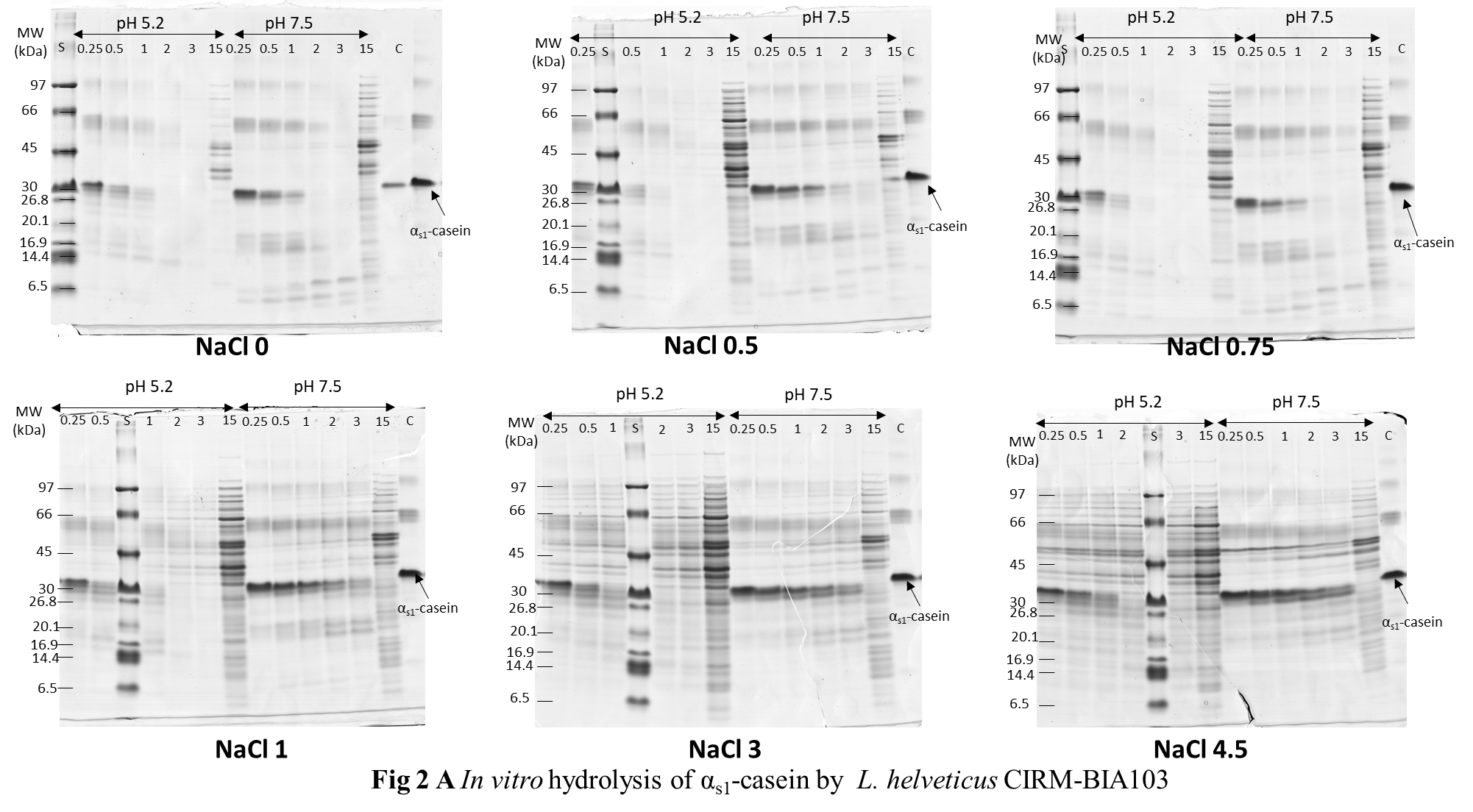


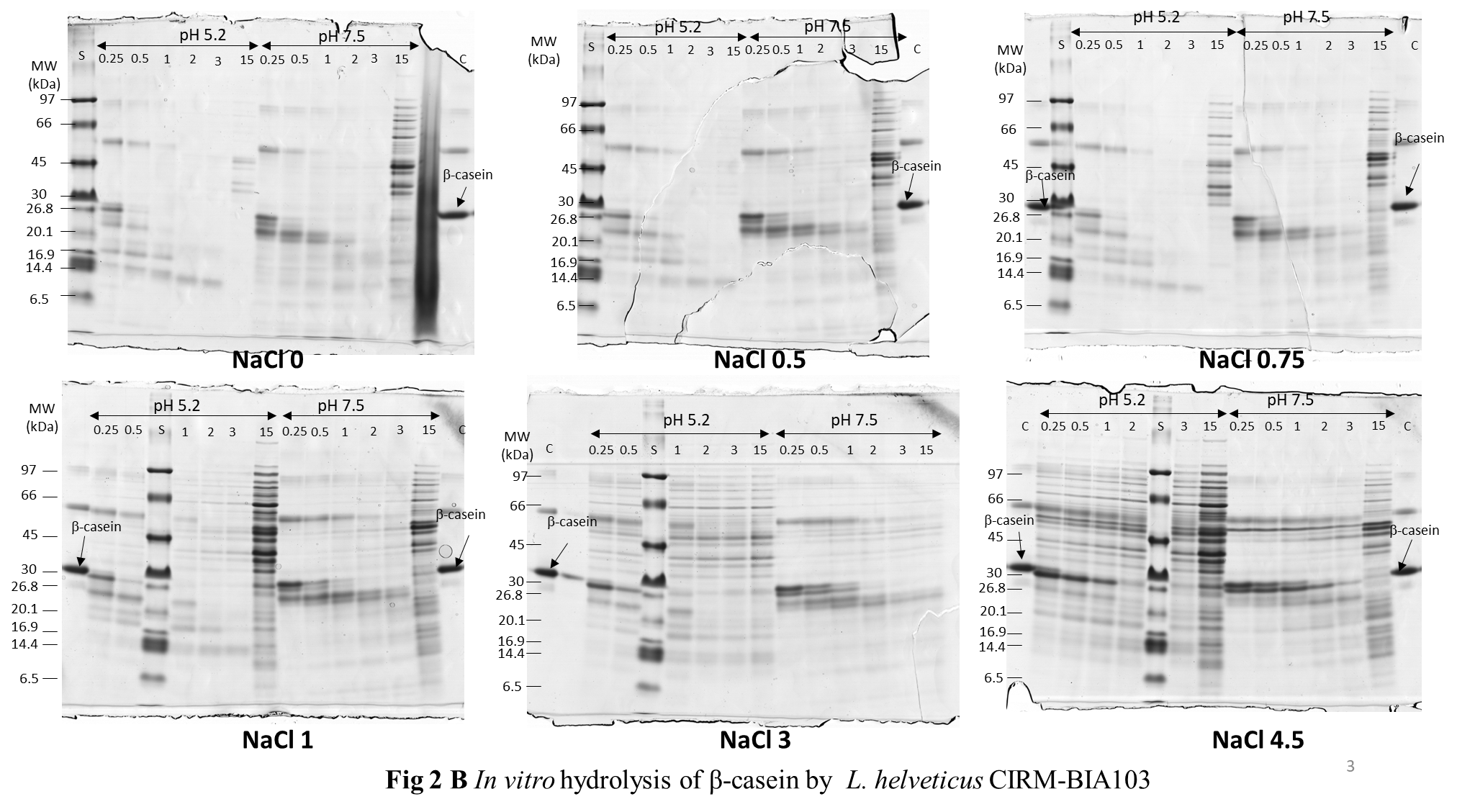


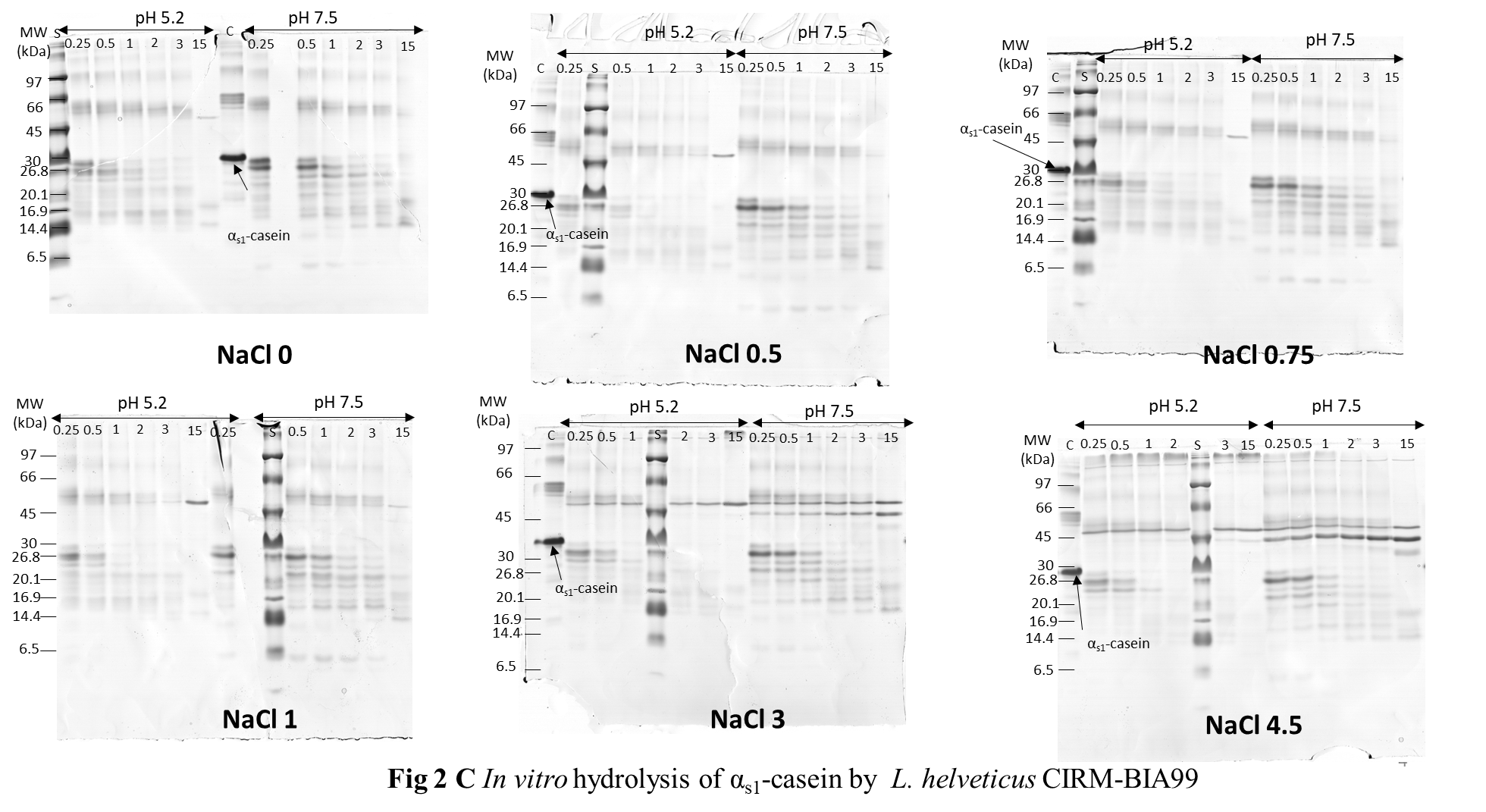


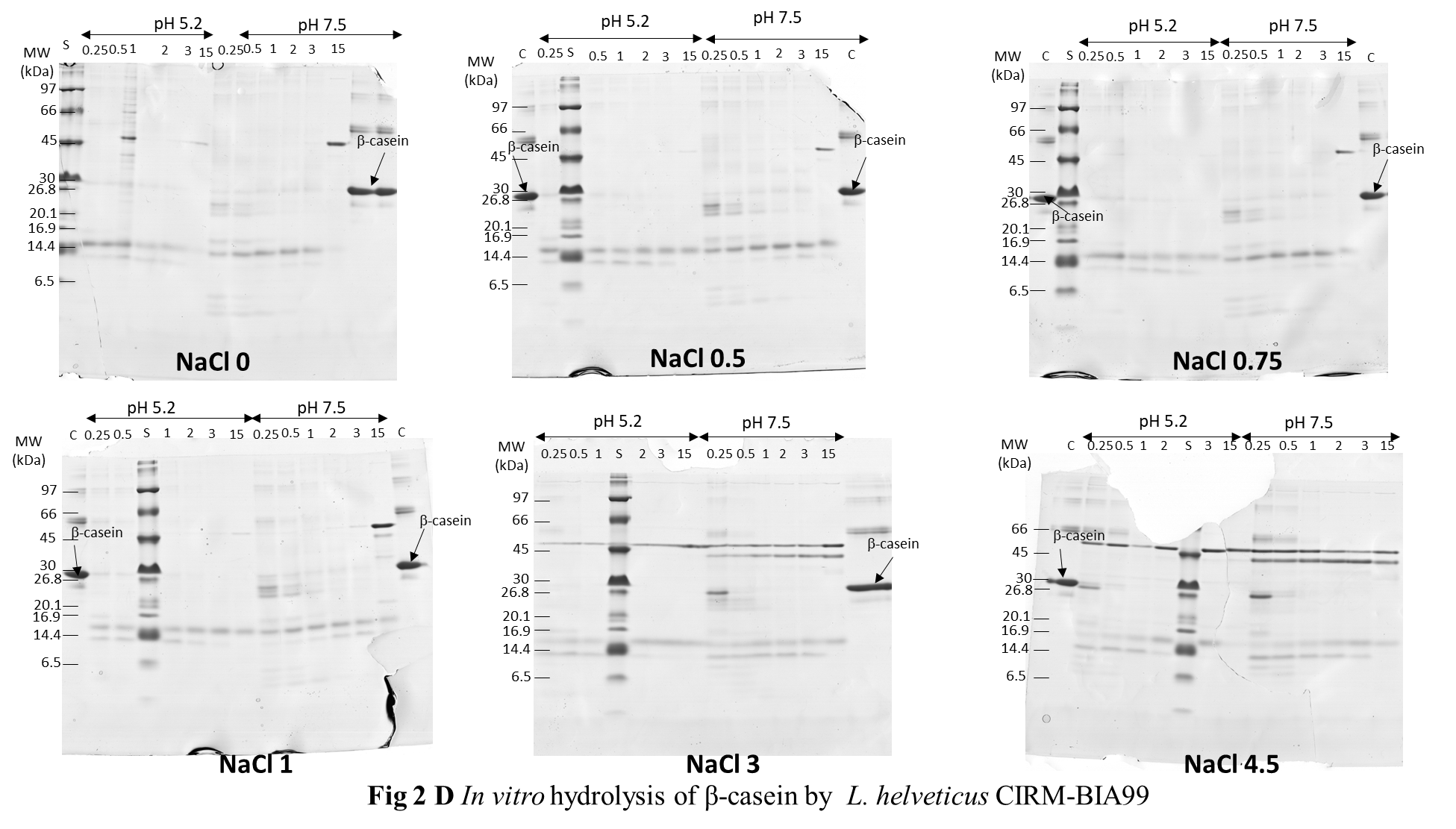


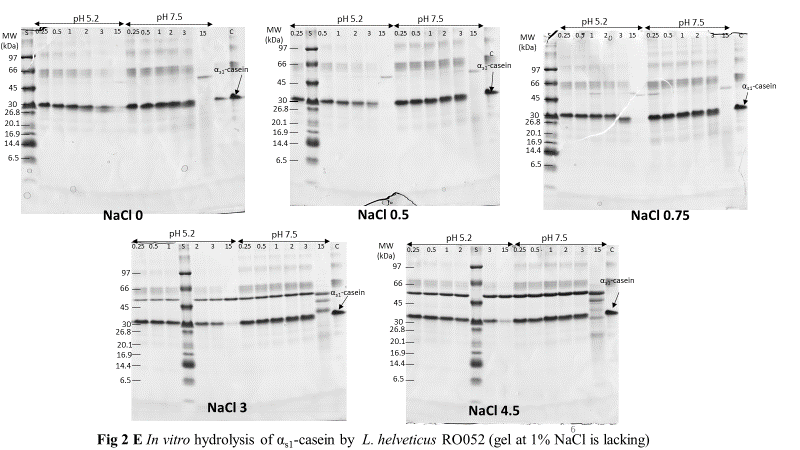


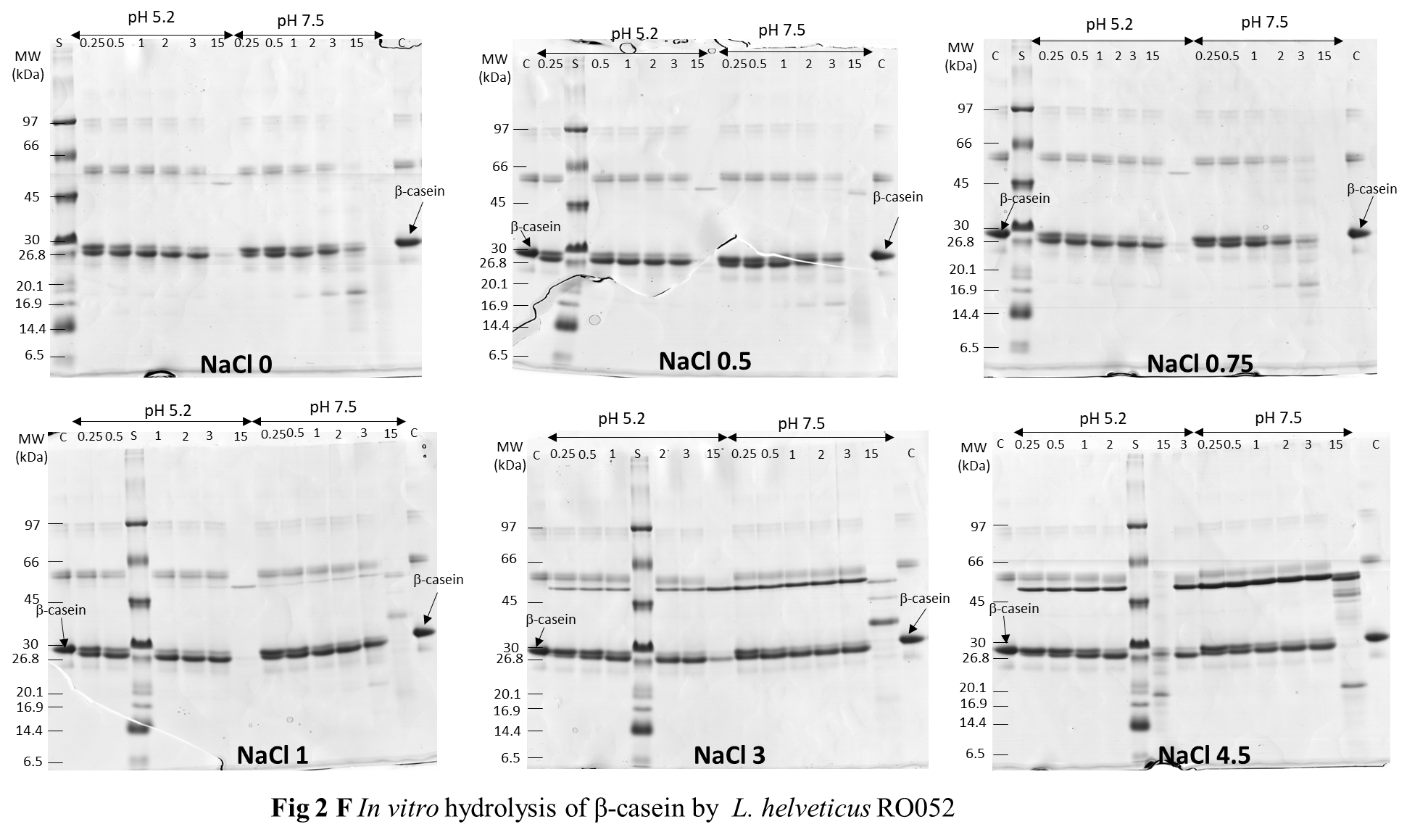

Supplement: Supplementary file 1 [file Data_Sheet_1.docx]
